# Supplementary material for: Clinical and DCE-CT signs in predicting microvascular invasion in cHCC-ICC
Source: Cancer Imaging. 2023 Nov 17;23:112. doi: 10.1186/s40644-023-00621-3 (PMC10655417; doi:10.1186/s40644-023-00621-3)
Supplement: Supplementary file 2 — Supplementary Material 2 [file 40644_2023_621_MOESM2_ESM.docx]

**Clinical and DCE-CT signs in predicting microvascular invasion in cHCC-ICC**

Zhong-Jian Liao^1,#^, Lun Lu^2,#^, Yi-Ping Liu^2,#^, Geng-geng Qin^1,3^, Cun-geng Fan^1^, Yan-Ping Liu^1^, Ning-yang Jia^2,^*, Ling Zhang^1,3,^*

1. Medical Imaging Department of Ganzhou People’s Hospital, Ganzhou, 341000, China.

2. Department of Radiology, Shanghai Eastern Hepatobiliary Surgery Hospital, Second Military Medical University, Shanghai 200438, China.

3. Department of Radiology, Nanfang Hospital, Southern Medical University, Guangzhou 510515, China.

*#Zhong-Jian Liao and Lun Lu, Yi-Ping Liu contributed equally to this paper*

***Corresponding author**: 1.Ling Zhang, Email address: 41733348@qq.com, Tel: + 862062786561.

2. Ning-yang Jia, Email adress:jianingyang6@163.com, Tel: 86-02181875211

**Abstract**

**Background:** To predict the microvascular invasion (MVI) in patients with cHCC-ICC.

**Methods:** A retrospective analysis was conducted on 119 patients who underwent CT enhancement scanning (from September 2006 to August 2022). They were divided into MVI-positive and MVI-negative groups.

**Results**: The proportion of patients with CEA elevation was higher in the MVI-positive group than in the MVI-negative group, with a statistically significant difference (P=0.02). The MVI-positive group had a higher rate of peritumoral enhancement in the arterial phase (P=0.01) whereas the MVI-negative group had more oval and lobulated masses (P=0.04). According to the multivariate analysis, the increase in CEA (OR=10.15, 95% CI: 1.11, 92.48, p=0.04), hepatic capsular withdrawal (OR=4.55, 95% CI: 1.44, 14.34, p=0.01) and peritumoral enhancement (OR=6.34, 95% CI: 2.18, 18.40, p<0.01) are independent risk factors for predicting MVI. When these three imaging signs are combined, the specificity of MVI prediction was 70.59% (series connection), and the sensitivity was 100% (parallel connection).

**Conclusions**: Our multivariate analysis found that CEA elevation, liver capsule depression, and arterial phase peritumoral enhancement were independent risk factors for predicting MVI in cHCC-ICC.

**Keywords**: Combined hepatocellular intrahepatic cholangiocarcinoma; microvascular invasion; computerized tomography

**Key points**

1. CEA elevation, liver capsule depression, and arterial phase peritumoral enhancement were independent risk factors for predicting MVI in cHCC-ICC.
2. When these three imaging signs are combined, the specificity of MVI prediction was 70.59% (series connection), and the sensitivity was 100% (parallel connection).
3. **Background**

Combined hepatocellular intrahepatic cholangiocarcinoma (cHCC-ICC) is a rare subtype of primary liver cancer with an incidence of 0.4% - 14.2%^[1]^. The tumor contains components of hepatocellular carcinoma and cholangiocarcinoma, which may originate from the same kind of liver progenitor cells and differentiate in different directions^[2]^. However, some studies have shown that mature hepatocytes can dedifferentiate into progenitor cells under certain inducements, thus transforming into cHCC-ICC^[3]^. Prognostic factors affecting cHCC-ICC are similar to those affecting HCC, including tumor size, multiple lesions, portal vein invasion, microvascular invasion^[4]^, stage of BCLC, and treatment options such as operation type, TARE, and efficacy of systemic immunotherapy^[4-8]^. MVI is a part of the pathological results and is difficult to be identified by imaging inspection. The imaging signs that can prompt MVI have been analyzed and reported in numerous studies. It is hoped that MVI can be predicted before surgery to help clinicians develop reasonable and informative surgical plans. However, MVI studies mainly are focused on hepatocellular carcinoma (HCC), a more common subtype of liver cancer^[9-12]^. Only Wang et al. have reported MRI signs for predicting MVI in cHCC-ICC^[13, 14]^. No study has been found to predict MVI in cHCC-ICC with CT signs. Due to the complexity of the imaging signs of cHCC-ICC, we intended to make a preliminary exploration in this regard.

1. **Data and methods**
   1. Clinical data

Data of 151 cases of cHCC-ICC from patients who had received dynamic enhanced CT scanning in three hospitals from September 2006 to August 2022 were retrospectively collected.

Inclusion criteria: 1. Age 18$-$80 years old; 2. CT indicates liver tumor $\geq$ 1 cm; 3. Completed the surgical resection of liver tumor, which had been confirmed as cHCC-ICC by postoperative pathology.

Exclusion criteria: 1. Age $＜$18 years old or over 80 years old; 2. Poor quality of CT scan image or the phase was incomplete; 3. Those who did not receive resection of the liver tumor but only underwent puncture; 4. Incomplete laboratory test data; 5. Those who had received other treatments before the operation.

Data of levels of alpha-fetoprotein (AFP), carcinoembryonic antigen (CEA), carbohydrate antigen 199 (CA-199), alanine aminotransferase (ALT), aspartate aminotransferase (AST), PLT, total bilirubin (TBIL), and γ-glutamyl transpeptidase (GGT), as well as quantitative analysis results of hepatitis B virus were acquired.

This was a retrospective study approved by the hospital ethics committee. All images and clinical data were acquired from the PACS system; thus, informed consent was exempted.

2.2 CT examination Equipment 1: Siemens 64 slice CT scanner, scanning parameters: tube voltage 120 kV, tube current automatic mA regulation technology, layer thickness 5 mm; Reconstruction parameters: pitch 1, reconstruction layer thickness 1 mm. The window width was 250 Hu, and the window level was 50 Hu. The tracking monitoring started 10 s after the contrast medium was injected. When the CT value of the monitoring layer reached the trigger threshold, the early arterial phase was acquired at 6 s delay and the late arterial phase was obtained at 8 s delay. The portal vein phase was captured 60 s after injection of the intravenous contrast medium, and the delayed image was obtained 120 s after injecting the contrast medium. The images were transmitted to PACS in DICOM format.

Equipment 2: GE 64 slice CT scanner (Biograph m CT). Parameters: layer thickness 6 mm, voltage 120 kV, matrix 512×512. The hepatic artery phase was 20~30 s, the portal vein phase was 50~60 s, the delay phase was 180~200 s, the window width was 250~300 HU, and the window level was 40~80 HU.

2.3 Analysis of CT signs Two radiologists who have been engaged in abdominal imaging diagnosis for more than 15 years analyzed the signs of all cHCC-ICC cases in the PACS system, including whether there was atrophy of the liver lobe, depression of the liver capsule, dilation of the surrounding bile duct, size, edge, shape of the focus, the presence or absence of capsule and lipid inside, CT density, tumor thrombus in the portal vein, enhancement methods in each phase of enhanced scanning, whether there was a peritumoral enhancement in the arterial phase. The evaluators had no prior knowledge of patients’ clinical data. In case of doubt, consultation would be initiated to reach an agreement.

2.4 Pathological analysis Microvascular invasion (MVI) refers to the microscopic observation of cancer cell nests in the vascular lumen lined with endothelial cells, mainly portal vein or hepatic vein branches (including intrathecal vessels). Pathological grading method: M0: no MVI was found; M1 (low-risk group): ≤ 5 MVI, and it occurred in the liver tissue near the cancer; M2 (high-risk group): >5 MVI, or MVI occurring in the liver tissue near the distant cancer. After reading the images, it was found that there were only a few M2 cases in this group, and thus M1 and M2 were combined into the MVI-positive group.

An experienced pathologist (who has 16-year experience in the pathological diagnosis of liver cancer) was asked to review the sections of all cases and evaluate whether cirrhosis was present. The relationship between CT findings and histopathology was jointly determined by radiologists and pathologists.

Specimens were fixed with 4% neutral buffered formaldehyde and embedded in conventional paraffin μM serial sections, routine HE staining and immunohistochemical staining. DAB (3,3'-diaminobenzidine) was used in immunohistochemical (IHC) staining. Hematoxylin was applied for re-staining, prior to which the slides were hydrated. Blue flower was dehydrated and sealed. Factor Ⅷ related antigen (F - Ⅷ Ag) was used to display the microvessels in the lesions.

1. **Results**

3.1 Clinical characteristics

See Table 1 for details. After screening, there were 119 cases of cHCC-ICC with complete CT plain scan and enhancement data and confirmed by surgery and pathology, which met the inclusion criteria, with a mean age of 52.41 ± 10.36, and a male-to-female ratio of 105:14. There was no significant difference between the MVI-positive group and negative group in age, sex, tumor size, lymph node metastasis, hepatitis B and cirrhosis (P>0.05). Laboratory examination: The proportion of patients with CEA elevation was higher in the MVI-positive group than in the MVI-negative group, with a statistically significant difference (*P* < 0.05). There was no significant difference in AFP, CA-199, ALT, AST, PLT, TBIL, or r-GT between the MVI-positive group and the MVI-negative group of cHCC-ICC (P>0.05).

3.2 CT plain scan and dynamic enhanced scan characteristics

See Table 1 for details. The MVI-positive group of 119 patients with cHCC-ICC was significantly different from the MVI-negative group in peripheral enhancement and tumor shape at the arterial phase (*P*$＜$0.05). MVI-positive group had a higher rate of peritumoral enhancement in the arterial phase whereas MVI-negative group had more oval and lobulated masses (P=0.04). No statistical differences were observed between the two groups in terms of imaging signs such as capsule, intravenous tumor thrombus, tumor density, margin, internal lipid composition, hepatic affinity, hepatic capsular regression, intravascular duct differentiation, schedule of reinforcement, and dynamic enhancement curve.

Risk factor analysis: See Table 2 for details. According to the multivariate analysis, the increase in CEA (OR=10.15, 95% CI: 1.11, 92.48, p=0.04), hepatic capsular withdrawal (OR=4.55, 95% CI: 1.44, 14.34, p=0.01) and peritumoral enhancement (OR=6.34, 95% CI: 2.18, 18.40, p<0.01) are independent risk factors for predicting MVI.

The three imaging characteristics that have the value of predicting MVI - CEA elevation, sensitivity, and specificity, i.e., PPV and NPV of the hepatic capsular withdrawal and peritumoral enhancement, as well as their combination to predict MVI are shown in Table 3. When these three imaging signs are combined, the specificity of MVI prediction was 70.59% (series connection), and the sensitivity was 100% (parallel connection).

1. **Discussion**

Predicting MVI in patients with primary liver cancer has been a hot research topic in the past decade because it is closely related to postoperative recurrence and metastasis and affects disease-free survival and overall survival time of patients. High-risk factors related to MVI in HCC include tumor diameter, number, AFP level, arterial phase annular enhancement, arterial phase peritumoral enhancement, etc.^[14-16]^ Whether MVI plays a key role in the prognosis of patients with ICC is still unclear. Studies that are focused on MVI in ICC are rarely seen and only a few reports are comprehensive^[17, 18]^. Age, γ-glutamyl transpeptidase, preoperative tumor number, larger tumor diameter, and intrahepatic bile duct dilatation may be independent risk factors for predicting MVI in ICC.

CHCC-ICC has the tumor tissue components of both HCC and ICC, so its imaging signs are complex and changeable. When HCC was the main component, the enhancement mode of enhanced scanning was similar to that of HCC, with obvious enhancement in the arterial phase and rapid clearance in the venous phase and delayed phase; With ICC as the main component, the arterial phase showed marginal enhancement, and the venous phase and delayed phase showed progressive centripetal enhancement^[19, 20]^. A few research reports on preoperative MVI prediction of cHCC-ICC^[13, 14]^ found that high AFP level (>400 ng/mL), arterial phase peritumoral enhancement, multiple nodules, γ-glutamyl transpeptidase could be independent risk factors for predicting MVI. However, the risk factors reported in those studies are not completely the same. In addition, when there is fat deposition in the tumor, it is a protective factor for MVI, which is similar to other studies that predict MVI in HCC^[21, 22]^. The prognosis of HCC is better when there is fat in the tumor on MRI, with longer disease-free survival and total survival.

As we all know, CEA is a tumor marker for colorectal cancer and other adenocarcinomas, and has recently attracted much attention as a potential marker for ICC: Compared with moderately differentiated ICC, CEA increases are more often seen in poorly differentiated ICC, and the 1-year and 5-year survival of patients with CEA increases are shorter^[23, 24]^. This suggests that it is a tumor marker of poor prognosis in ICC. Our data show that the increase of CEA is positively correlated with the positive MVI in cHCC-ICC, but whether there is a causal relationship between these two indicators is unknown. Hepatic capsular retraction refers to the localized depression of the smooth envelope of the liver, which was once considered to be related to liver malignancy. Many benign lesions and treatments can cause hepatic capsular retraction^[25]^. It may indicate the potential process of liver fibrosis or focal atrophy of liver parenchyma caused by vascular injury/chronic biliary inflammation and may explain the cHCC-ICC in the more aggressive MVI-positive group, which is more likely to lead to vascular and bile duct injury around the focus. Another reason may be that the proportion of our cases located in the periphery of the liver was too high. It has been supported by a large number of literature^[10, 26-28]^ that MVI in HCC can be predicted by the arterial phase peritumoral enhancement. It has also been confirmed that it can be used to predict the MVI in cHCC-ICC, a rare subtype of primary liver cancer.

Due to the rarity of this tumor, its clinicopathological features are still not well understood. The surgical resection plan, indication for liver transplantation, and the corresponding adjuvant treatment plan are still under exploration. More clinical and imaging studies on cHCC-ICC will be conducted in the future. We can do a better job in using CT, MRI, and other imaging techniques to predict the efficacy of various treatment regimens and forecast the disease-free survival and overall survival of patients with this disease.

Limitations: Our cases were from three hospitals and the CT machines used for scanning were different from the scanning parameters, which may lead to inconsistent interpretation of some image signs. In addition, we did not extract the image omics features in this study but used manual film reading, which would have certain limitations and subjectivity. In the next step, we will add the image omics features to predict MVI before surgery and increase the sample size to make the prediction model more accurate. Besides, our research used CT as an imaging tool, whose effect of observing lipid components with small structures in the tumor is not as good as MRI. We will collect more cases of cHCC-ICC undergoing MRI examination for an accurate preoperative diagnosis and predict its therapeutic efficacy and survival.

1. **Conclusion**

Our multivariate analysis found that CEA elevation, liver capsule depression, and arterial phase peritumoral enhancement were independent risk factors for predicting MVI in cHCC-ICC.

**Abbreviations**

cHCC-ICC: Combined hepatocellular intrahepatic cholangiocarcinoma

MVI: microvascular invasion CEA: Carcinoembryonic antigen

**AFP: alpha-fetoprotein** DCE: dynamic contrast enhanced

## Declarations

**Ethics approval and consent to participation**

The retrospective study was approved by the ethics committee of Ganzhou People’s Hospital, and the requirement for signed informed consent was waived. We confirm that all methods were performed in accordance with the relevant guidelines.

**Consent for publishing**

Not applicable.

**Availability of data and materials**

Data to replicate findings are in the Figures and Tables of the main paper. Due to patient privacy protection, any additional materials of the study are only available upon individual request directed to the corresponding author.

**Competing interests**

The authors declare that there is no conflict of interest regarding the publication of this paper.

**Funding**

This work was supported by the Natural Science Foundation of Guangdong Province (2018A030313951), the science and technology plan project of Ganzhou (2022--RC1339, 2022--RC1349).

**Author’s contributions**

ZJL, LL, and YPL participated in the study design, evaluated the results, and wrote the first and revised manuscript. CGF and YPL participated in the study design and provided the contrast medium. LZ, GGQ, and NYJ carried out the image analysis and revised manuscripts. LZ participated in the design of the study and redesigned the data analysis. All authors read and approved the final manuscript.

**Acknowledgments**

### We would like to thank the whole study team at our Hospitals, for continuous support.

Figure legends:

Fig. 1 cHCC-ICC presenting with MVI in a 48-year-old man. (A) A plain CT scan showed an irregular low-density mass; (B-D) Early enhancement of the mass was observed during the arterial phase of CT enhancement, followed by rapid clearance of the contrast agent from the mass. Hepatic capsule retraction (green arrow) and peritumoral enhancement (red arrow) were observed.

Fig. 2 cHCC-ICC presenting with MVI in a 53-year-old woman. (A) A plain CT scan revealed a large low-density mass in the right lobe of the liver, capsule retraction can be seen around the mass (green arrow). (B-D) The arterial phase of the enhanced CT scan showed significantly uneven enhancement and peritumoral enhancement (red arrow).

**参考文献**

[1] Seehawer M, D'Artista L, Zender L. The Worst from Both Worlds: cHCC-ICC[J]. Cancer Cell, 2019,35(6):823-824.

[2] Xue R, Chen L, Zhang C, et al. Genomic and Transcriptomic Profiling of Combined Hepatocellular and Intrahepatic Cholangiocarcinoma Reveals Distinct Molecular Subtypes[J]. Cancer Cell, 2019,35(6):932-947.

[3] Rosenberg N, Van Haele M, Lanton T, et al. Combined hepatocellular-cholangiocarcinoma derives from liver progenitor cells and depends on senescence and IL-6 trans-signaling[J]. J Hepatol, 2022,77(6):1631-1641.

[4] Wakizaka K, Yokoo H, Kamiyama T, et al. Clinical and pathological features of combined hepatocellular-cholangiocarcinoma compared with other liver cancers[J]. J Gastroenterol Hepatol, 2019,34(6):1074-1080.

[5] Di Federico A, Rizzo A, Carloni R, et al. Atezolizumab-bevacizumab plus Y-90 TARE for the treatment of hepatocellular carcinoma: preclinical rationale and ongoing clinical trials[J]. Expert Opin Investig Drugs, 2022,31(4):361-369.

[6] Rizzo A, Cusmai A, Gadaleta-Caldarola G, et al. Which role for predictors of response to immune checkpoint inhibitors in hepatocellular carcinoma?[J]. Expert Rev Gastroenterol Hepatol, 2022,16(4):333-339.

[7] Santoni M, Rizzo A, Kucharz J, et al. Complete remissions following immunotherapy or immuno-oncology combinations in cancer patients: the MOUSEION-03 meta-analysis[J]. Cancer Immunol Immunother, 2023,72(6):1365-1379.

[8] Rizzo A, Ricci A D, Brandi G. Systemic adjuvant treatment in hepatocellular carcinoma: tempted to do something rather than nothing[J]. Future Oncol, 2020,16(32):2587-2589.

[9] Tang Y, Xu L, Ren Y, et al. Identification and Validation of a Prognostic Model Based on Three MVI-Related Genes in Hepatocellular Carcinoma[J]. Int J Biol Sci, 2022,18(1):261-275.

[10] Xu X, Zhang H L, Liu Q P, et al. Radiomic analysis of contrast-enhanced CT predicts microvascular invasion and outcome in hepatocellular carcinoma[J]. J Hepatol, 2019,70(6):1133-1144.

[11] Lv K, Cao X, Du P, et al. Radiomics for the detection of microvascular invasion in hepatocellular carcinoma[J]. World J Gastroenterol, 2022,28(20):2176-2183.

[12] Surov A, Pech M, Omari J, et al. Diffusion-Weighted Imaging Reflects Tumor Grading and Microvascular Invasion in Hepatocellular Carcinoma[J]. Liver Cancer, 2021,10(1):10-24.

[13] Wang X, Wang W, Ma X, et al. Combined hepatocellular-cholangiocarcinoma: which preoperative clinical data and conventional MRI characteristics have value for the prediction of microvascular invasion and clinical significance?[J]. Eur Radiol, 2020,30(10):5337-5347.

[14] Huang S S, Zuo M X, Xie C M. Combining Preoperative Clinical and Imaging Characteristics to Predict MVI in Hepatitis B Virus-Related Combined Hepatocellular Carcinoma and Cholangiocarcinoma[J]. J Pers Med, 2023,13(2).

[15] Yang L, Gu D, Wei J, et al. A Radiomics Nomogram for Preoperative Prediction of Microvascular Invasion in Hepatocellular Carcinoma[J]. Liver Cancer, 2019,8(5):373-386.

[16] Zhang L, Yu X, Wei W, et al. Prediction of HCC microvascular invasion with gadobenate-enhanced MRI: correlation with pathology[J]. Eur Radiol, 2020,30(10):5327-5336.

[17] Chen Y, Liu H, Zhang J, et al. Prognostic value and prediction model of microvascular invasion in patients with intrahepatic cholangiocarcinoma: a multicenter study from China[J]. BMC Cancer, 2021,21(1):1299.

[18] Qian X, Lu X, Ma X, et al. A Multi-Parametric Radiomics Nomogram for Preoperative Prediction of Microvascular Invasion Status in Intrahepatic Cholangiocarcinoma[J]. Front Oncol, 2022,12:838701.

[19] Chen J, He J, Deng M, et al. Clinicopathological, radiologic, and molecular study of 23 combined hepatocellular-cholangiocarcinomas with stem cell features, cholangiolocellular type[J]. Hum Pathol, 2017,64:118-127.

[20] Choi J H, Ro J Y. Combined Hepatocellular-Cholangiocarcinoma: An Update on Pathology and Diagnostic Approach[J]. Biomedicines, 2022,10(8).

[21] Siripongsakun S, Lee J K, Raman S S, et al. MRI detection of intratumoral fat in hepatocellular carcinoma: potential biomarker for a more favorable prognosis[J]. AJR Am J Roentgenol, 2012,199(5):1018-1025.

[22] Min J H, Kim Y K, Lim S, et al. Prediction of microvascular invasion of hepatocellular carcinomas with gadoxetic acid-enhanced MR imaging: Impact of intra-tumoral fat detected on chemical-shift images[J]. Eur J Radiol, 2015,84(6):1036-1043.

[23] Moro A, Mehta R, Sahara K, et al. The Impact of Preoperative CA19-9 and CEA on Outcomes of Patients with Intrahepatic Cholangiocarcinoma[J]. Ann Surg Oncol, 2020,27(8):2888-2901.

[24] Loosen S H, Roderburg C, Kauertz K L, et al. CEA but not CA19-9 is an independent prognostic factor in patients undergoing resection of cholangiocarcinoma[J]. Sci Rep, 2017,7(1):16975.

[25] Panick C, Ward R D, Coppa C, et al. Hepatic capsular retraction: An updated MR imaging review[J]. Eur J Radiol, 2019,113:15-23.

[26] Chong H H, Yang L, Sheng R F, et al. Multi-scale and multi-parametric radiomics of gadoxetate disodium-enhanced MRI predicts microvascular invasion and outcome in patients with solitary hepatocellular carcinoma </= 5 cm[J]. Eur Radiol, 2021,31(7):4824-4838.

[27] Hong S B, Choi S H, Kim S Y, et al. MRI Features for Predicting Microvascular Invasion of Hepatocellular Carcinoma: A Systematic Review and Meta-Analysis[J]. Liver Cancer, 2021,10(2):94-106.

[28] Chen J, Ming X, Wang Z, et al. Analysis of the Performance of Gadoxetic Acid Disodium MRI in Predicting Microvascular Invasion of Hepatocellular Carcinoma[J]. Contrast Media Mol Imaging, 2022,2022:6128845.
