# Supplementary material for: Clinical and DCE-CT signs in predicting microvascular invasion in cHCC-ICC
Source: Cancer Imaging. 2023 Nov 17;23:112. doi: 10.1186/s40644-023-00621-3 (PMC10655417; doi:10.1186/s40644-023-00621-3)
Supplement: Supplementary file 1 — Supplementary Material 1 [file 40644_2023_621_MOESM1_ESM.docx]

**Revision Notes**

Dear Editor:

Thank you for your letter and for the reviewers’comments concerning ours manuscript entitled “*Clinical and DCE-CT signs in predicting microvascular invasion in cHCC-ICC*”(Submission ID: CAIG-D-23-00275). Those comments are all valuable and very helpful for revising and improving our paper, as well as the important guiding significance to our researches. We have studied comments carefully and have made correction which we hope meet with approval. The redundant portion are deleted in strikethrough in the paper. The main corrections in the paper and the responds to the reviewer’s comments are as following:

Responds to the reviewer’s comments:

Reviewer #1:

**1. The reviewer’s comment:** Introduction section: although the authors correctly included important papers in this setting, we believe the systemic treatment scenario for HCC should be further discussed in the introduction section and some recently published studies should added (  PMID: 35403533; PMID: 34798793; PMID: 36633661; PMID: 32772560 ), only for a matter of consistency. We think it might be useful to introduce the topic of this interesting study.

**The authors’ Response**: Thank you for your advice. Indeed, factors affecting the prognosis of HCC and cHCC-ICC are not only MVI, tumor volume, multiple lesions, portal vein invasion, but also clinical stage, treatment choice, etc. We have reviewed these references. I have a new understanding of the treatment plan of liver cancer, and I have added the quotation in the introduction of this article. Next year, our radiology department may cooperate with clinical departments to try to evaluate the response of HCC patients to immunotherapy with CT and MRI. This is a good idea, and we look forward to communicating with each other in the next article.

2. **The reviewer’s comment:** The authors should further discuss the limitations of the current paper, including the sample size.

**The authors’ Response**: Thanks for your suggestions, we have added the shortcomings of the research and the direction of future research in the section of ***Limitions.***

3. **The reviewer’s comment:** The authors should also include a more personal perspective to reflect on. For example, they could answer the following questions - in order to facilitate the understanding of this complex topic to readers: What are the knowledge gaps and how do researchers tackle them? How do you see this area unfolding in the next 5 years? We think it would be extremely interesting for the readers.

**The authors’ Response**: Your suggestion has given me new inspiration and promoted me to persist in the road of studying cHCC-ICC for a longer time. I have included the limitations of the current study and the outlook for the future in the penultimate paragraph of the discussion. And I believe that more scholars will join this field and deepen their understanding of it.

Reviewer #2:

1. **The reviewer’s comment:**

Overall the manuscript is well organized, crisp in content and with sound methodology.

Following modifications may be suggested;-Limitations need to be addressed in more detail (such as effect of sample size and utility of MRI)

**The authors’ Response**: Thank you very much for your recognition of my research. I improved the ***limitation*** part as you suggested.

2. **The reviewer’s comment:** Recent management recommendations / alternatives to surgical treatment may be taken into consideration.

**The authors’ Response**: Since this study is about MVI for preoperative prediction of cHCC-ICC, the treatment plans of the cases have not been collected, but recently I have read some literatures on the treatment of cHCC-ICC, and found that I can predict the efficacy of various treatment plans with imaging omics, which will be my next research direction, thank you very much for your suggestion.

3. **The reviewer’s comment:** Clarification on terminologies used such as hepatic capital involvement and perimetric enhancement.

**The authors’ Response**: I feel very sorry for my negligence. Word errors have been fixed.
